# Chronic Wasting Disease Drives Population Decline of White-Tailed Deer

**DOI:** 10.1371/journal.pone.0161127

**Published:** 2016-08-30

**Authors:** David R. Edmunds, Matthew J. Kauffman, Brant A. Schumaker, Frederick G. Lindzey, Walter E. Cook, Terry J. Kreeger, Ronald G. Grogan, Todd E. Cornish

**Affiliations:** 1 Department of Veterinary Sciences, University of Wyoming, Laramie, Wyoming, United States of America; 2 United States Geological Survey, Wyoming Cooperative Fish and Wildlife Research Unit, Department of Zoology and Physiology, University of Wyoming, Laramie, Wyoming, United States of America; 3 College of Agriculture and Natural Resources, University of Wyoming, Laramie, Wyoming, United States of America; 4 Wyoming Game and Fish Department, Wheatland, Wyoming, United States of America; University of Arkansas Fayetteville, UNITED STATES

## Abstract

Chronic wasting disease (CWD) is an invariably fatal transmissible spongiform encephalopathy of white-tailed deer, mule deer, elk, and moose. Despite a 100% fatality rate, areas of high prevalence, and increasingly expanding geographic endemic areas, little is known about the population-level effects of CWD in deer. To investigate these effects, we tested the null hypothesis that high prevalence CWD did not negatively impact white-tailed deer population sustainability. The specific objectives of the study were to monitor CWD-positive and CWD-negative white-tailed deer in a high-prevalence CWD area longitudinally via radio-telemetry and global positioning system (GPS) collars. For the two populations, we determined the following: a) demographic and disease indices, b) annual survival, and c) finite rate of population growth (*λ*). The CWD prevalence was higher in females (42%) than males (28.8%) and hunter harvest and clinical CWD were the most frequent causes of mortality, with CWD-positive deer over-represented in harvest and total mortalities. Survival was significantly lower for CWD-positive deer and separately by sex; CWD-positive deer were 4.5 times more likely to die annually than CWD-negative deer while bucks were 1.7 times more likely to die than does. Population *λ* was 0.896 (0.859–0.980), which indicated a 10.4% annual decline. We show that a chronic disease that becomes endemic in wildlife populations has the potential to be population-limiting and the strong population-level effects of CWD suggest affected populations are not sustainable at high disease prevalence under current harvest levels.

## Introduction

In large mammals, chronic disease often manifests as having low detectability, moderate impacts on adult mortality and fecundity, and depressed population growth rates that are sensitive to changes in adult survival [1]. Chronic diseases are difficult to detect due to lack of mass mortalities, rapid population declines, or shifts in age structure [1]. Few studies have investigated population-level impacts of chronic diseases in wildlife populations, despite the recently increasing interest and emphasis of population-level effects of wildlife diseases [2]. The dearth of well-studied population-level effects of chronic diseases is worrisome given that research suggests diseases with preclinical stages rather than acute diseases are more likely to influence long-term population-level dynamics [2]. The widespread potential of population-level impacts warrants further research on chronic wildlife diseases [2].

Confounding the issue of investigating chronic diseases is the temptation of, and pressure on, managers to react to newly discovered diseases in ways that may not be optimal. Chronic disease may have minor effects on population vital rates early in a disease epidemic [3]. However, if the disease state shifts from epidemic to endemic, then vital rates may not be affected for years or decades and monitoring must be completed over an extended time-frame. Monitoring is crucial because a firm understanding of the effects of disease on population vital rates is necessary to accurately model disease dynamics and determine suitable management options [3]. Unfortunately, wildlife diseases are challenging to study because of their insidious nature, logistical difficulties, statistical challenges, and high costs [2, 3]. This is the case for chronic wasting disease of white-tailed deer (*Odocoileus virginianus*), mule deer (*Odocoileus hemionus*), Rocky Mountain elk (*Cervus elaphus nelsoni*), and moose (*Alces alces shirasi*) [4–8].

Chronic wasting disease (CWD) is a uniformly fatal, progressive neurodegenerative transmissible spongiform encephalopathy (TSE) that occurs in wild cervid populations in 21 U.S. states and two Canadian provinces [9]. The TSEs are caused by proteinase-resistant, abnormal isoforms (PrP^res^) of normal host cellular proteins (PrP^C^) known as prions. The causative agent of CWD is known as PrP^CWD^.

There are few studies on population-level effects of CWD on cervid populations. One such study was conducted on a mule deer population near Boulder, Colorado, USA [10]. Deer abundance declined 45% during 1988–2006. It was believed CWD had been endemic since 1985 and was highly prevalent (males = 41%; females = 20%). The decline was attributed to high prevalence of CWD resulting in low overall adult survival (0.72).

Information suggests CWD has potential to cause population declines and possibly localized extinctions at high prevalence; however, this has not been definitively proven or observed. To address if and how CWD negatively impacts deer population dynamics, we intensively monitored a white-tailed deer population in southeastern Wyoming over a protracted time-period (2003–2010) to estimate population vital rates and model the influence of disease on population performance. We hypothesized that demographic rates are altered by CWD to an extent large enough to lower the population growth rate. The specific objectives were to monitor CWD-positive and CWD-negative white-tailed deer in a high-prevalence CWD endemic area throughout their lifespan via radio-telemetry and global positioning system (GPS) collars. We sought to determine the following for the two segments of the population: a) demographic and disease indices including CWD prevalence, causes of mortality, pregnancy and recruitment rates, b) annual survival, and c) finite rate of population growth (*λ*). These indicators allowed us to determine the magnitude of the effect of CWD on a free-ranging white-tailed deer population.

## Materials and Methods

Anesthesia was used on all white-tailed deer that were captured and processed for enrollment into study. Deer were chemically immobilized with 0.03 mg/kg carfentanil and 0.7 mg/kg xylazine. All deer were injected subcutaneously with procaine/benzathine penicillin G combination (25,000 units/kg based on benzathine fraction, Bimeda, Le Sueur, Minnesota, USA) and intramuscularly with 1.5 mg/kg of Banamine (Intervet Inc., Merck Animal Health, Summit, New Jersey, USA). Immobilized deer were reversed with 100 mg naltrexone per 1 mg carfentanil and 2 mg/kg tolazoline and monitored until recovered and ambulatory. All animal procedures were approved through the University of Wyoming (Laramie, Wyoming, USA) Institutional Animal Care and Use Committee (Protocol #A-3216-01). Wyoming Game and Fish Department approved our Chapter 33 Capture Permit to capture the pre-determined number of white-tailed deer annually (Permit #531).

### Study System

The study was conducted primarily on the VR ranch (True Ranches, Casper, Wyoming, USA) and surrounding areas southwest of Glenrock, Wyoming (42.861N 105.871W) in southern Converse County (S1 Fig). Elevation ranged from 1,700 m in the lower plains to 2,000 m in the rolling to steep foothills. Deer Creek and its tributaries were the main habitat for white-tailed deer [11, 12]. Riparian habitat was dominated by cottonwood (*Populus* sp.), boxelder (*Acer negundo*), willow (*Salix* sp.), Rocky Mountain maple (*Acer glabrum*), serviceberry (*Amelanchier* sp.), and choke cherry (*Prunus virginiana*). Agricultural crops were comprised of grass hay (*Bromus* sp., *Dactylis* sp., *Phleum* sp.) and alfalfa (*Medicago sativa*). Natural draws and breaks surrounding agricultural fields were dominated by sagebrush (*Artemisia* spp.) and grassland communities. Higher elevations supported mountain mahogany (*Cercocarpus montanus*), ponderosa pine (*Pinus ponderosa*), and juniper (*Juniperus* sp.). Availability of natural forage and agricultural crops was plentiful and not limiting. Primary predators in the area included cougars (*Puma concolor*), coyotes (*Canis latrans*), black bears (*Ursus americanus*), and golden eagles (*Aquila chrysaetos*). Predator-caused mortality was rare in this population (see below) despite all four predators being relatively common. Cougars appeared to prefer mule deer as their target species in this study area (Cornish and DeVivo, unpublished data). There were approximately 19.2 deer/linear kilometer of riparian habitat while the surrounding habitats were sparsely populated.

This area is endemic for CWD in white-tailed deer, mule deer, and elk. The prevalence of CWD in white-tailed deer harvested in the surrounding Wyoming Game and Fish Department (WGFD) hunt area (65) was 32% in 2003 and 43% in 2010, and 33% (*n* = 132) overall during the study period (2003–2010; WGFD, unpublished data). These prevalence estimates were obtained from CWD testing hunter-killed deer randomly sampled and testing by enzyme-linked immunosorbent assay (ELISA) and immunohistochemistry (IHC) of retropharyngeal lymph nodes and/or obex region of the medulla oblongata. Importantly, the annual prevalence estimates vary quite dramatically due to small sample sizes using this method; however, the 33% prevalence based on an 8 year average likely is a good representation of the true population prevalence in adult (≥1.5 years old) white-tailed deer. Hunt area 65 is part of the historic CWD core area of SE Wyoming that has been tested routinely for presence of CWD since 1998; first year white-tailed deer were sampled was 1999 and the prevalence was 28.6% (4/14). It is not known how long CWD has been endemic, but it likely has occurred since the 1970’s. The WGFD did not actively manage for CWD in hunt area 65 during the study period other than annual surveillance of hunter-killed deer to track prevalence. The WGFD does not gather population data on white-tailed deer to set population objectives and they were hunted liberally within the hunt area during this period; however, hunting was not used to actively manage for CWD. Conversely, mule deer were hunted conservatively during this period due to poor population performance, possibly linked to high CWD prevalence.

### Field and Laboratory Methods

White-tailed deer were captured using Clover traps [13] and helicopter net-gunning (Leading Edge Aviation, Lewis, Idaho, USA; Quicksilver Air, Peyton, Colorado, USA) [14]. All marked deer were recaptured annually to test for CWD, replace collars or battery packs of GPS collars, and download data from GPS collars. Deer were chemically immobilized with 0.03 mg/kg carfentanil and 0.7 mg/kg xylazine based on Kreeger and Arnemo [15] and adjusted by T.C. Deer were fitted with either an ear tag (fawn—≤8 months) or collar containing a very high frequency (VHF) radio transmitter (Advanced Telemetry Systems, Inc., Isanti, Minnesota, USA). A subset of deer were collared with store-on-board GPS receivers (Lotek Wireless, Inc., Newmarket, Ontario, Canada) equipped with VHF transmitters during 2006–2009. Body condition scores were assessed on a scale of 0–5 based on palpating abdominal and rump subcutaneous fat deposits. Blood samples were collected by jugular venipuncture for pregnancy testing females. Tonsil biopsies for CWD testing were performed as described by Wolfe et al. [16]. Immobilized deer were reversed with 100 mg naltrexone per 1 mg carfentanil and 2 mg/kg tolazoline and monitored until recovered and ambulatory [17]. All animal procedures were approved through the University of Wyoming (Laramie, Wyoming, USA) Institutional Animal Care and Use Committee (Protocol #A-3216-01).

All collared deer, including GPS-collared deer because we were not able to remotely download data due to store-on-board technology, were monitored by radio telemetry for mortality status at least twice per week. In the event of mortality, the site was investigated for evidence of cause of death and dead deer were subjected to complete necropsies to determine cause of death. All carcasses were subjected to thorough CWD examinations, which involved IHC examination of tonsil, retropharyngeal lymph node, and obex region of the medulla oblongata. Necropsies and laboratory testing of pertinent samples collected during necropsy were performed at the Wyoming State Veterinary Laboratory, Laramie, Wyoming, USA. Based on telemetry and GPS data, deer were classified as migratory if winter and summer ranges did not overlap and as a disperser if they irreversibly moved to and occupied an area geographically distinct and non-overlapping of natal range [18]. Analyses for these procedures have been described previously [12].

Number of fawns at side of collared deer was determined in late August 2008 and early September 2009. The location was determined for collared deer using radio-telemetry triangulation from roads. Deer were approached on foot, displaced from day beds, and presence or absence of fawns was determined by observing the does fleeing with fawns at side. Fawns were approximately 2-months old at time of recruitment determination and were no longer staying hidden separate from their dams.

Tissue samples available to test for CWD included annual tonsil biopsies and whole tonsil, retropharyngeal lymph nodes, and medulla oblongata sectioned at the obex from carcasses depending on post-mortem condition. Tissues were examined by IHC by staining for PrP^CWD^ using monoclonal antibody F99/97.6.1 [19] and hematoxylin for counter-staining as described previously [20]. One ml serum samples from all female deer were tested for pregnancy-specific protein B [21] by BioTracking LLC (Moscow, Idaho, USA) to establish pregnancy status.

### Data Analyses

All statistical analyses and regression models were programmed using SAS (SAS Institute, Cary, North Carolina, USA) unless stated otherwise. We wished to determine the influence of covariates, including CWD-status, on the probability of pregnancy, which is an important vital rate to understand the population dynamics as it relates to CWD. We used PROC LOGISTIC to perform a logistic regression analysis [22] on probability of pregnancy (event = 1) given CWD-status (CWD), age, body condition score (BCS) at time of capture, and year of capture (year). No fawn (≤8 months) deer were pregnant, so that age class was excluded from analysis. Single parameter models were generated to begin forward parameter selection; however, none of the parameters were significant. We generated the full model containing all four parameters:

$$LOGIT\;\left(Pregnancy\right)=\beta_{0}+\beta_{1}CWD+\beta_{2}Age+\beta_{3}BCS+\beta_{4}Year$$ (1)

Pregnancy is an important vital rate used in the matrix population model, and thus we needed to determine if pregnancy varied by CWD-status to inform how to use the metric in the population matrix. Due to small sample sizes, we utilized PROC FREQ to perform Fisher’s exact *χ*^2^ analysis [23] comparing observed proportion of pregnant does to expected proportion of pregnant does by CWD-status for each capture year as an overall test for significance of CWD effects on pregnancy.

We needed to calculate fecundity estimates separately by CWD-status to determine if CWD impacted ability of does to raise fawns (a hypothesis of interest to population dynamics related to CWD) as well as to include as a vital rate in the population model. We performed a 2-group *t*-test [24] using PROC TTEST to compare average number of fawns per doe between CWD-positive and CWD-negative female deer. Recruitment was analyzed separately between 2008 and 2009. We used these same data with PROC GLIMMIX to perform a mixed model [25] given that some of the fawn counts were from the same does in 2008 and 2009. The model produced a log odds of fawn production given CWD-status, age, year, CWD x age, and CWD x year:

$$GLIMMIX\;\left(Fawn\;Count\right)=\beta_{0}+\beta_{1}CWD+\beta_{2}Age+\beta_{3}Year+\beta_{4}CWD\times Age+\beta_{5}CWD\times Year$$ (2)

To better understand what factors influence annual survival of white-tailed deer, we analyzed annual survival data using Cox proportional hazards model to examine survival differences given the following covariates: CWD-status, sex, age class, year, and migratory/dispersal status (binary) [26, 27]. We determined mortality dates as the first mortality event recorded by the GPS unit (4 hour delay) or estimated based on carcass condition from the first date of hearing a mortality signal during radio-telemetry for VHF-marked deer (4 or 6 hour delay depending on model). Deer were right censored at the date of the last relocation if lost to follow-up due to transmitter failure, dropped transmitter, or long-range dispersal and we failed to relocate with aerial telemetry. We also right censored deer killed during capture or by poachers, or that survived to the end of the study period. Deer killed legally by hunter harvest were not right censored as hunting was an integral part of the study system. Deer that initially were CWD-negative then tested CWD-positive during subsequent captures were right censored as CWD-negative at the capture date of first CWD-positive test. We tested proportionality of hazards ratios using the TEST option in PROC PHREG [28]. We utilized an Extended Cox model after we determined that proportionality of hazards ratios was not met (${Wald\;\chi }_{4}^{2}$ = 9.0252, *P* = 0.0605). We used PROC PHREG in SAS to evaluate the effects of the above covariates plus age x duration interaction term (to account for lack of proportionality) on annual survival of deer, modeled as duration known alive (duration) x living status (e.g., alive (0) or dead (1); status) [29]. The following was the full model:

$$PHREG\;\left(Duration\times Status\right)=\beta_{0}+\beta_{1}CWD+\beta_{2}Sex+\beta_{3}CWD\times Sex+\beta_{4}Age+\beta_{5}Age\times Log\left(Duration\right)+\beta_{6}Migration+\beta_{7}Dispersal+\beta_{8}Year$$ (3)

We implemented backwards elimination for parameter selection and Akaike’s Information Criteria (AIC) [30] for model selection (supported models were within 2 AIC values of the model with the lowest AIC value; ΔAIC) along with consideration of significant parameters (based on Wald *χ*^2^ statistic) and our biological knowledge of the system.

We wished to compare subcategory-specific annual survival rates based on capture year_*t*_ to capture year_*t*+1_ to better understand the pairwise comparisons of significant factors from the Cox proportional hazards modeling. While the Cox proportional hazards modeling indicates which factors are important and the risk associated with each factor, it does not provide an actual survival estimate to be used for intra- and inter-population comparisons. Therefore, we used Kaplan-Meier survival estimation [31] to generate these metrics with PROC LIFETEST in SAS. We generated survival estimates separately by age and CWD-status for males and females. The *χ*^2^ Log-Rank test [32] determined differences in annual survival rates by sub-categories using PROC LIFETEST by strata.

We also needed to generate age and CWD-status specific survival estimates by biological year (June 1 –May 31) for does to be used as a vital rate in the matrix population model. For these analyses, we once again used the Kaplan-Meier survival estimator. Biological year of fawns was defined as September 1 –May 31 because fawn recruitment of marked does was determined during the first week in September. However, these fawns were not marked with radio transmitters; we captured fawns when they were much older in February; therefore, we had to estimate fawns survival from September to February. We combined published estimates of fawn survival during this missing time period from Dusek et al. [33] and survival data of fawns tracked on this study from February1 through May 31 with a weighted average to account for differing lengths of time between the two sources of survival estimates to produce one fawn survival estimate.

We needed to calculate annual CWD incidence as a vital rate to be used within the matrix population model, but we also needed to perform comparisons on these estimates by sex and age class to inform how to populate the matrix with this metric. We calculated annual CWD incidence using the time-to-event (CWD conversion) Kaplan-Meier estimator [31] with PROC LIFETEST. Incidence was calculated separately by sex and for each age-class. We performed a 2-group *t*-test [24] to determine if a difference in incidence existed between bucks and does using PROC TTEST (Cochran option).

**Fig 1:**
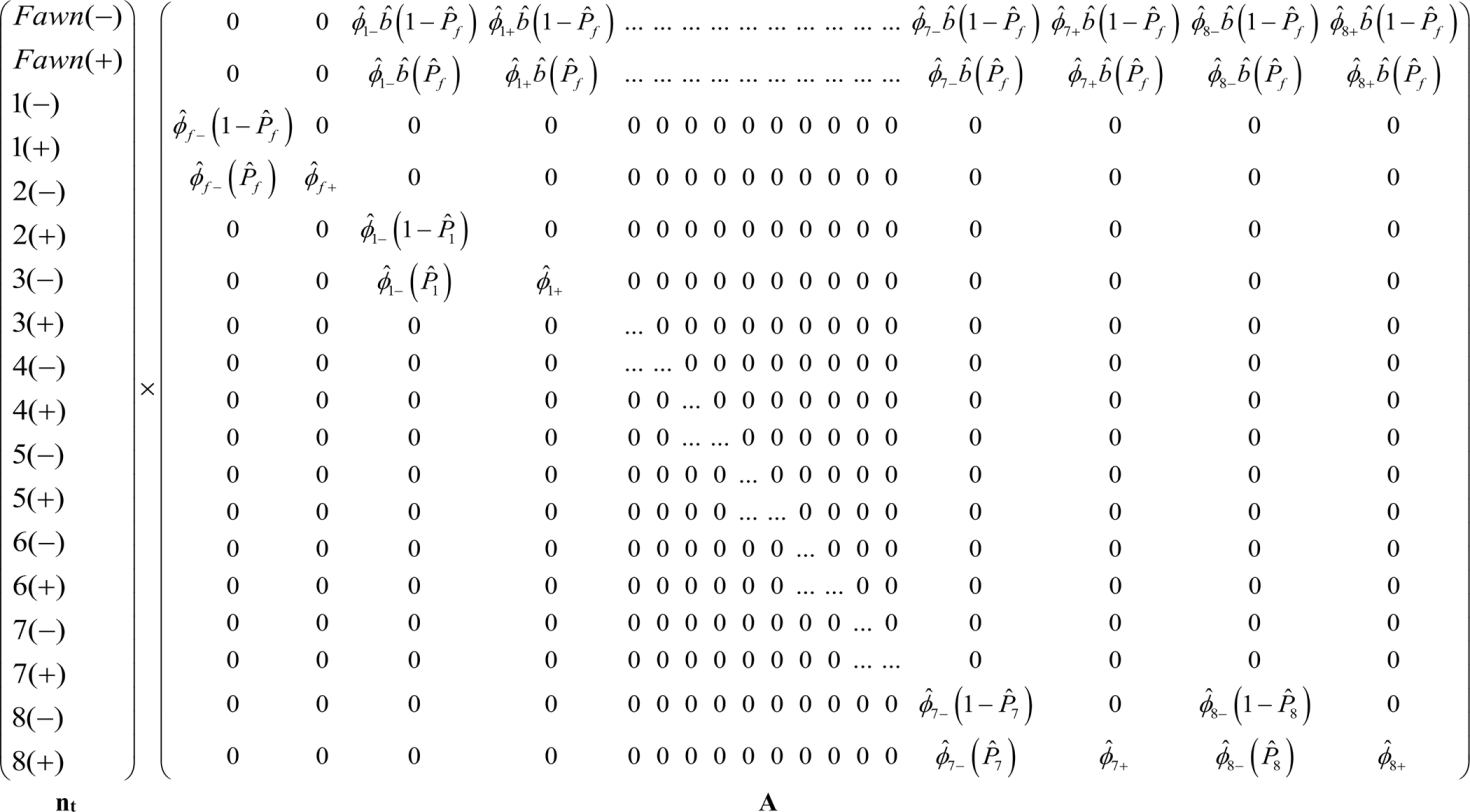
Leslie Matrix Population Model. Post-breeding, age-structured, female-dominated 18x18 Leslie Matrix model of white-tailed deer population on Deer Creek drainage SW of Glenrock, WY (2003–2010) that was located within the chronic wasting disease (CWD) endemic area. n_t_ represents the number of deer in each age class by CWD-status ((-) = PrP^CWD^ not detected, (+) = PrP^CWD^ detected). ${\hat{\phi }}_{i(-/+)}$ represents estimated survival by age class, *i*, and CWD-status (- or +), $\hat{b}$ is the estimated fecundity rate and ${\hat{P}}_{i}$ is the age-specific CWD incidence rate.

Our ultimate question of interest was to determine the impact of CWD on the growth rate of this population. We calculated the finite rate of population growth (*λ*) using a post-breeding, age-structured, female-only 18 x 18 dimension Leslie matrix [34, 35] in MATLAB^®^ (The MathWorks, Inc., Natick, Massachusetts, USA). Vital rates incorporated were fecundity (average number of fawns per doe in the first week of September) and age-specific pregnancy rates, survival rates, and CWD incidence. All vital rates were estimated separately for CWD-negative and CWD-positive deer except fecundity, which did not differ by CWD-status. The 18 x 18 transition matrix, **A**, represented the estimated demographic rates of the study population with both CWD-negative and CWD-positive females, and the transition between them due to infection, represented (Fig 1). We calculated the population growth rate as the dominant eigenvalue, *λ*_1_ [35]. To determine the population vital rates that most influenced lambda (i.e., the vital rate that lambda was most sensitive to and would change the most if those vital rates changed), we performed sensitivity analysis of **A** in MATLAB using the *vitalsens*.*m* function developed by Morris and Doak [36] to quantify how sensitive *λ* was to a change in value of each vital rate. In addition, we determined the elasticity of each vital rate [35].

We calculated the 95% confidence interval for *λ*_1_ using parametric bootstrapping [36]. Specifically, 2000 each of age and CWD-status specific survival and CWD incidence rates were randomly estimated from the *β*-distribution using the *betaval* function and 2000 estimates of the fecundity rate were generated using the *stretchbetaval* function; both functions were from the *popbio* package [37] in program R v.3.2.5 [38]. The estimates were based on the mean and bias-corrected variance estimate generated using the *Kendall* function in the *popbio* package. We then used the 2000 vital rate estimates to generate 2000 *λ*_1_ estimates with the *eigen*.*analysis* function in the *popbio* package based on the **A** matrix and we determined the 95% confidence interval from the 0.025 and 0.975 quantiles of the *λ*_1_ distribution [36]. The *popbio* package reproduces the same results in program R as the MATLAB-based analysis.

To understand the effect of CWD on population vital rates, we created two matrices, **Aneg** and **Apos,** taking the form of 9 x 9 transition matrices for CWD-negative and CWD-positive populations respectively. The two matrices differed in that one model assumed 0% CWD-prevalence and one model assumed 100% CWD-prevalence, allowing determination of the magnitude in change of *λ* (Δλ) due to CWD. We performed a life table response experiment (LTRE) on the transition matrices, **Aneg** and **Apos,** using the *vitalsens*.*m* function in MATLAB [35] to better understand the influence of CWD on *λ*_1_.

To evaluate the influence of CWD incidence on population growth rate, we varied incidence from 0 to 1.0 by 0.05. This range of incidence rates was inserted in the full 18 x 18 matrix model, keeping all other parameters equal, and calculating *λ* across the range. State wildlife agencies routinely track CWD by annual prevalence from hunter-harvested or targeted deer and elk. We converted each incidence rate into annual prevalence based on the following equation:

$$P=\frac{I\times \bar{D}}{1+I\times \bar{D}}$$ (4)

where *P* was prevalence, *I* was incidence, and $\bar{D}$ was the estimated duration of illness estimated by Kaplan-Meier [31] using PROC LIFETEST. All CWD-positive deer were included in the analysis with the enrollment date set as the date of first positive CWD test and mean time known alive (i.e., $\bar{D}$) calculated by Kaplan-Meier analysis [31].

We were interested in the population age structure to determine if the population was shifted to a young age structure. We calculated the dominant right eigenvector (**w**_**1**_), which gives the stable population structure, using function *eigenall*.*m* in MATLAB [35] to determine the stable population age structure. Given the following equation:

$$Aw_{i}=\lambda_{i}w_{i}$$ (5)

where **w**_***i***_’s are age-specific contributions to population growth. When one sums the age specific **w**_***i***_’s and then divides each **w**_***i***_ by the total, the proportion of the population in each age class is determined [35]. The age structure of both male and female deer by CWD-status was determined using this method.

## Results

During the study period (January 2003–February 2010), 112 deer were captured as fawns (≤8 months-old; female: male = 57: 55) and 63 deer captured originally as adults (≥1.5 years-old; female: male = 27: 36). All deer were recaptured annually. Overall CWD prevalence during the study period (last known CWD-status of each individual deer) was 35.4% (*n* = 161). Prevalence was higher in does (42%, *n* = 81) than bucks (28.8%, *n* = 80, $\chi_{1}^{2}$ = 6.608, *P* = 0.01). Average annual CWD prevalence (based on annual tonsil biopsies) was 23.8% (*n* = 345) overall, 24.3% (*n* = 202) for does, and 23.1% (*n* = 143) for bucks.

There were 118 mortalities (CWD-negative = 64, CWD-positive = 50, CWD-unknown = 4) during the study period (S1 Table). Hunter harvest was the most common cause of mortality (*n* = 46) and more CWD-positive deer (*n* = 19) were harvested than expected based on average annual CWD prevalence (41.3% vs. 23.8%, $\chi_{1}^{2}$ = 8.876, *P* = 0.029). Bucks were more common (76%) than does in the harvest. There were 20 capture-related mortalities, representing 4.2% of all captures (*n* = 476). This is an overestimate of capture-related mortality because many non-target deer were captured and released without injury during Clover trapping. Seventeen deer (female: male = 12: 5) died of clinical CWD; does comprised 71% of clinical cases, but made up only 48% of the study population.

Age, CWD-status, year, and body condition score did not influence pregnancy. Average proportion pregnant for CWD-negative deer was 0.95 (*n* = 109, 95% C.I. = 0.92–0.99) and for CWD-positive deer was 0.92 (*n* = 38, 95% C.I. = 0.84–1.0). There was not a statistically significant difference detected in proportion pregnant by CWD-status annually or across all years combined ($\chi_{1}^{2}$ = 0.601, *P* = 0.438).

Average number of fawns per doe was 0.74 (95% C.I. = 0.47–1.00). There was no statistically significant difference detected in the average number of fawns per doe by CWD-status in 2008 (CWD-negative = 0.56, CWD-positive = 0.67, *t*_17_ = -0.23, *P* = 0.819) or 2009 (CWD-negative = 0.90, CWD-positive = 1.00, *t*_13_ = -0.22, *P* = 0.829).

Six Cox proportional hazards models were evaluated using AIC, statistical significance of parameter estimates, and biological knowledge of the system. The full model included the following parameters: CWD, Sex, CWD x Sex, Age, Age x Time, Migration, Dispersal, and Year; the top model included CWD, Sex, Age, Age x Time, and Dispersal. The most significant parameter was CWD, which had the highest hazard ratio. The CWD-positive deer were 4.51 times more likely to die annually than CWD-negative deer (*β*_1_ = 1.51, $\chi_{1}^{2}$ = 44.62, *P* <0.001, 95%, C.I. = 2.9–7.0). Bucks were 1.70 times more likely to die than does (*β*_2_ = 0.532, $\chi_{1}^{2}$ = 5.17, *P* = 0.023, 95% C.I. = 1.08–2.69). Deer that did not disperse were 1.61 times more likely to die than deer that did disperse; however, the result was not statistically significant (*β*_5_ = -0.493, $\chi_{1}^{2}$ = 1.118, *P* = 0.290, 95% C.I. = 0.656–4.08). Age and age over time did not affect survival probability.

Kaplan-Meier survival log rank tests were performed on all ages combined (overall) and by each age class (Table 1). Survival comparisons were all statistically different except CWD-positive females vs. males ($\chi_{1}^{2}$ = 2.73, *P* = 0.098), indicating sex did not significantly affect survival of CWD-positive deer. Of the five significant comparisons, CWD-positive deer had lower survival than CWD-negative deer and males had lower survival than females. Log rank tests were highly significant by CWD-status and less-so by sex, which indicated CWD was a greater indicator of annual survival. There were no clear trends by age class.

**Table 1:** Annual Survival Rate Comparisons.

Category	Results	Overall	Fawn	1.5	2.5	3.5	4.5	5.5+
**Female:**	Survival: CWD (-)	0.853	0.552	0.889	0.875	0.741	1.00	1.00
**CWD (-) vs. (+)**	Survival: CWD (+)	0.481	*n* = 1	0.400	0.500	0.500	0.500	0.500
	$\chi_{1}^{2}$	23.49	---	8.99	0.875	4.12	5.33	1.69
	*P*-value	**<0.001**	---	**0.003**	0.351	**0.042**	**0.021**	0.194
**Male:**	Survival: CWD (-)	0.729	0.332	0.791	0.667	0.525	1.00	---
**CWD (-) vs. (+)**	Survival: CWD (+)	0.304	*n* = 1	0.200	0.200	0.500	0.333	---
	$\chi_{1}^{2}$	13.53	---	9.11	6.05	0.000	4.09	---
	*P*-value	**0.000**	---	**0.003**	**0.014**	1.00	**0.043**	---
**All deer:**	Survival: CWD (-)	0.801	0.737		0.750	0.780	1.00	1.00
**CWD (-) vs. (+)**	Survival: CWD (+)	0.396	0.250		0.333	0.500	0.429	0.444
	$\chi_{1}^{2}$	39.70	17.33		9.52	3.73	8.90	10.21
	*P*-value	**<0.001**	**<0.001**		**0.002**	0.054	**0.003**	**0.001**
**All deer:**	Survival: Females	0.758	0.524	0.745	0.788	0.668	0.786	---
**Female vs. male**	Survival: Males	0.612	0.543	0.645	0.455	0.643	0.750	---
	$\chi_{1}^{2}$	11.96	1.14	0.514	6.03	2.03	0.520	---
	*P*-value	**0.001**	0.286	0.473	**0.014**	0.155	0.471	---
**CWD (-):**	Survival: Females	0.853	0.552	0.889	0.875	0.741	1.00	---
**Female vs. male**	Survival: Males	0.729	0.332	0.791	0.667	0.700	*n* = 1	---
	$\chi_{1}^{2}$	11.03	0.671	1.39	2.99	1.64	---	---
	*P*-value	**0.001**	0.413	0.239	0.084	0.200	---	---
**CWD (+):**	Survival: Females	0.433	0.333		0.500	0.500	0.500	---
**Female vs. male**	Survival: Males	0.304	0.167		0.200	0.500	0.333	---
	$\chi_{1}^{2}$	2.73	0.028		1.72	0.064	0.270	---
	*P*-value	0.098	0.868		0.190	0.800	0.604	---

Kaplan-Meier survival rates and log rank *χ*^2^ test results by sex and chronic wasting disease (CWD)-status (CWD-negative = (-), CWD-positive = (+)) of white-tailed deer captured, CWD-tested annually, radio-collared, and monitored by radio-telemetry SW of Glenrock, WY (2003–2010). Results presented overall and by age-classes.

**Fig 2:**
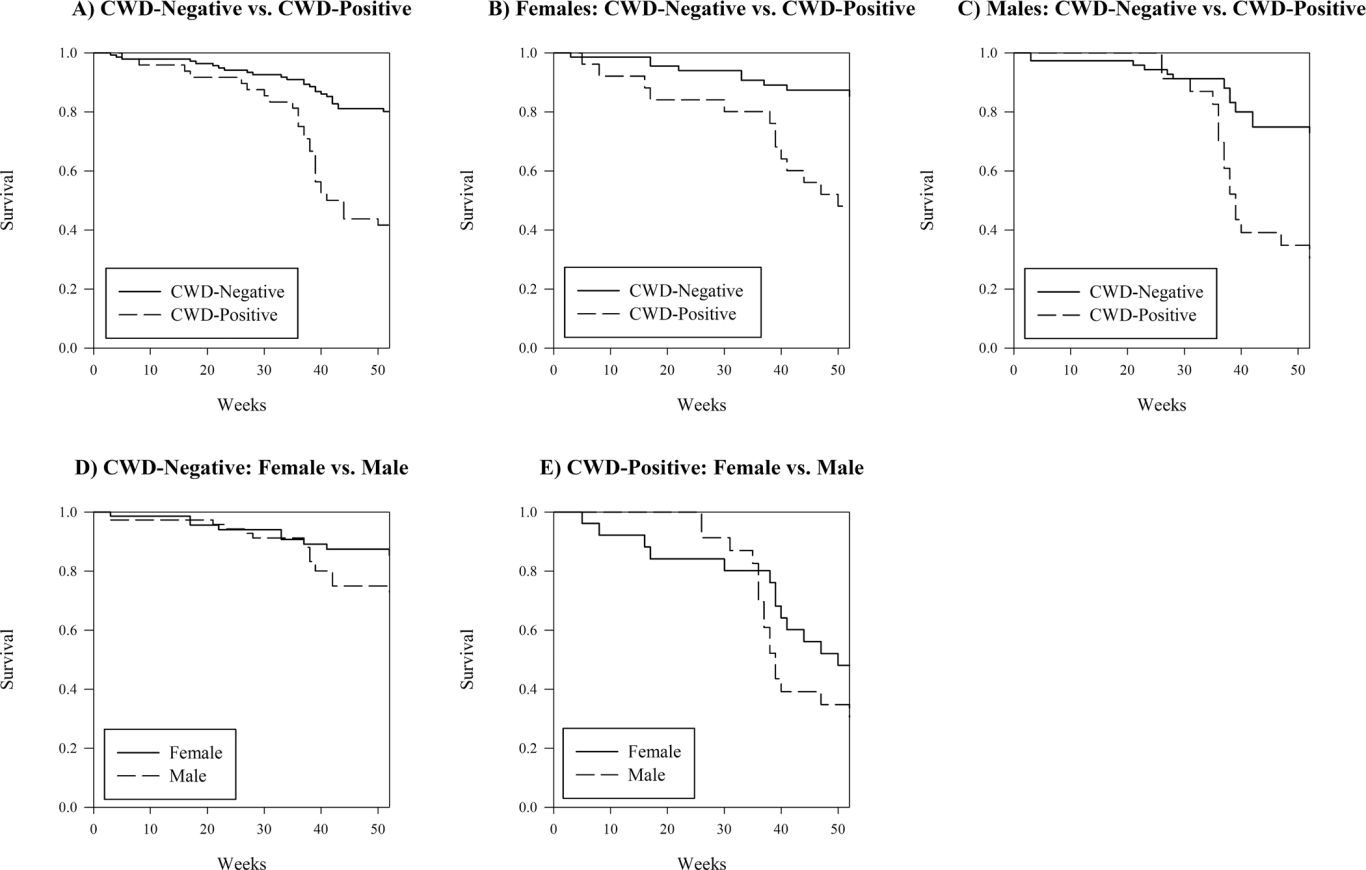
Kaplan-Meier Annual Survival Rate Curves. Survival rate curves of segments of the white-tailed deer study population that was captured, tested for CWD by tonsil biopsy, marked with radio transmitters, and followed by radio telemetry SW of Glenrock, WY (2003–2010).

There was a large difference in annual survival of CWD-positive deer (0.396) compared to CWD-negative deer (0.801, $\chi_{1}^{2}$ = 39.70, *P* = <0.001, Fig 2A). Kaplan-Meier survival curves indicated a slow, steady drop in survival of CWD-positive does (Fig 2B). Survival estimates were similar during most of the year for CWD-positive and CWD-negative bucks and then dropped slowly for CWD-negative bucks but precipitously for CWD-positive bucks between weeks 35–40, which coincided with the 6-week hunting season. Fewer CWD-negative bucks survived annually (73%) than CWD-negative does (85%; Fig 2D). The CWD-positive deer were the only group that did not differ significantly by sex, but both survival rates were extremely low (Fig 2E, Table 1).

**Fig 3:**
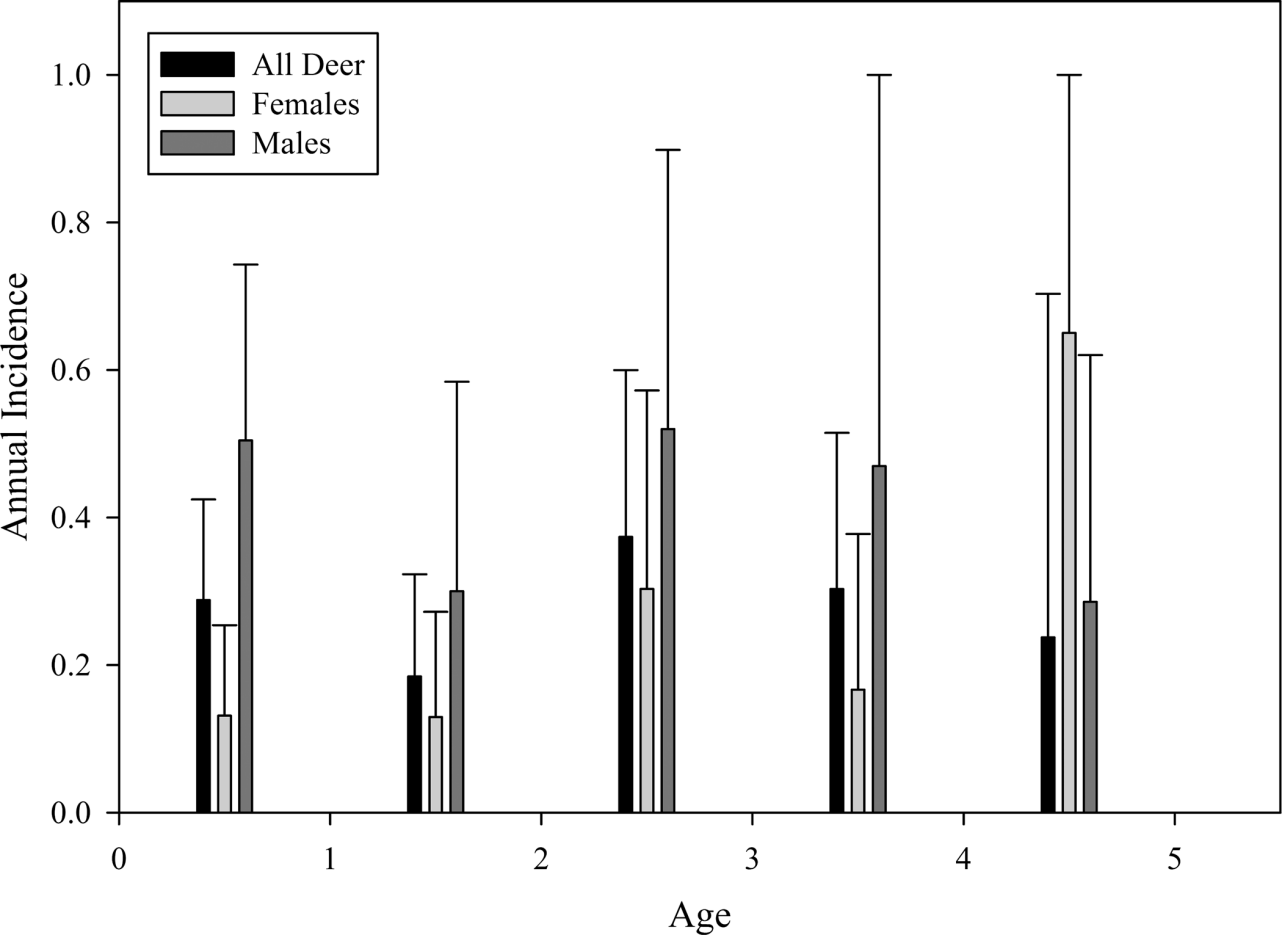
Annual Incidence Rates. Chronic wasting disease (CWD) annual incidence rate by sex and age class of white-tailed deer captured, CWD-tested annually, radio-collared, and monitored by radio-telemetry SW of Glenrock, WY (2003–2010). The CWD incidence was calculated by Kaplan-Meier time to event analysis.

Annual CWD incidence increased more rapidly in bucks, reaching peak in the first year of life (51%—indicated by CWD-positive test as a 1.5 years-old (yearling)), declining slightly in the second year, returning to near peak incidence during the third year, and then declining steadily to 0 by the 6^th^ year (Fig 3). Incidence increased slower in females, but reached a higher peak than males (65%) during the 5^th^ year, then also dropping to 0 during the 6^th^ year. Incidence was not significantly different between sexes (*t*_4_ = -1.26, *P* = 0.277).

The dominant eigenvalue, *λ*_1_, of our 18 x 18 matrix model was 0.896 (0.859–0.980), which indicates 10.4% annual decline from 2003–2010, assuming a stable age distribution. A *λ*_1_ of 0.896 is not sustainable (*t*_0.5_ = 5 years, *t*_extinction_ = 48 years). To determine magnitude of Δ*λ*_1_ due to CWD, *λ*_1_ was determined for a subpopulation of CWD-negative (**Aneg**) and CWD-positive (**Apos**) deer, which were 1.07 and 0.681 respectively. The results suggest CWD significantly depressed *λ*_1_ in the study population.

Sensitivity analysis indicated *λ*_1_ was most sensitive to changes in survival of CWD-negative fawns (0.280) and yearlings (0.269) and, to a lesser extent, 2.5 years-olds (0.154). Fecundity was not important; however, *λ*_1_ was slightly sensitive to changes in fecundity of yearlings (0.156). Changes to vital rates in older age classes did not significantly affect *λ*_1_. Elasticity results were similar to sensitivity analysis, except in the case of fecundity, which had small values (≤0.06) for every age class (S2 Table).

Survival of yearlings and 2.5 years-olds was most severely reduced by CWD and had the greatest impact on lowering *λ*_1_. These results, combined with sensitivity analysis, suggest that survival overall across younger age cohorts is influencing *λ*_1_. The LTRE indicated that survival of yearlings and 2.5 years-olds contributed most to the change in *λ*_1_ (Δ *λ*_1_) cause by CWD, with Δ *λ*_1_ of 0.144 and 0.173 respectively. No other vital rate caused a Δ *λ*_1_ ≥0.025. The LTRE was similar to sensitivity analysis except in the case of fawn survival, which contributed 0 to the treatment effect because survival of CWD-negative and CWD-positive fawns was equal. Overall treatment effect of CWD was 0.348. The magnitude of the negative effect on *λ*_1_ by CWD infection was 0.382. Survival was recalculated for each age class with all hunter-related mortalities right censored from Kaplan-Meier analysis. With hunting-related mortality censored, the resulting vital rates estimated a *λ*_1_ of 1.00.

**Fig 4:**
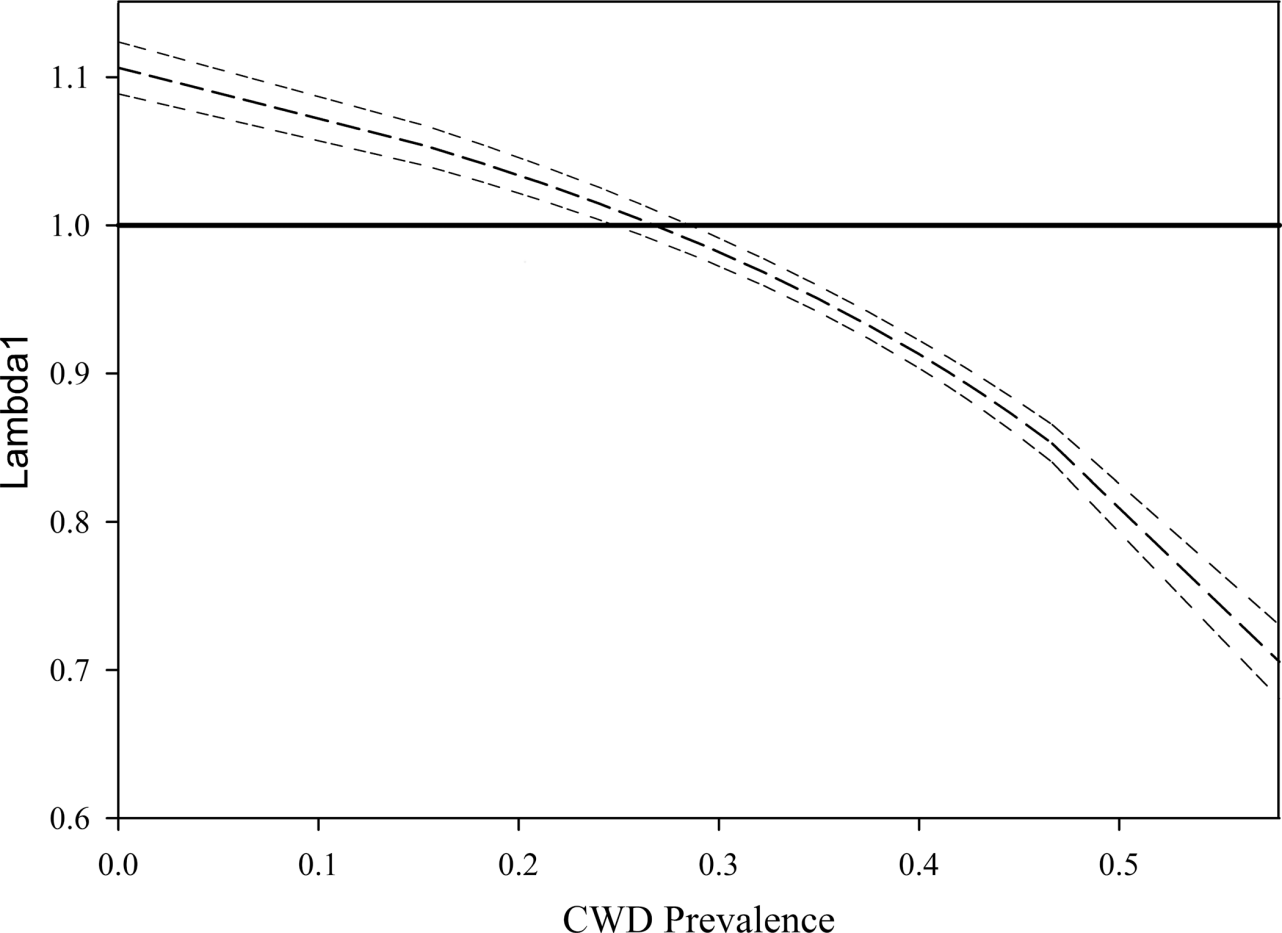
Lambda by Prevalence. Curvilinear relationship between increasing chronic wasting disease (CWD) prevalence and decreasing lambda (*λ*_1_) simulated from vital rates of a white-tailed deer population captured, CWD-tested annually, marked with radio transmitters, and monitored by radio-telemetry SW of Glenrock, WY (2003–2010). The curve was generated by holding all population vital rates constant, but varying incidence up and down from the population incidence by intervals of 0.05, re-running the Leslie matrix population model with the constant vital rates and altered incidence value populating the transition matrix, **A**, and calculating lambda. The incidence rates were then converted into prevalence estimates to be more useful to wildlife managers because state wildlife agencies collect surveillance data in prevalence proportions, not incidence rates. The solid horizontal line at *λ*_1_ = 1.0 represents the threshold at which population growth begins to decline (*λ*_1_< 1.0) and the dark dashed line is the simulated population growth rate with accompanying 95% confidence intervals (lighter double dashed lines).

By varying incidence rates, recalculating *λ*_1_ for each incidence rate and plotting *λ*_1_ by incidence, it was determined *λ*_1_ dropped below 1.0 at an annual incidence rate of 0.26. Transforming incidence into prevalence and then plotting the *λ*_1_ values with the new prevalence values estimated *λ*_1_ was <1.0 at 0.27 (27%, Fig 4).

**Fig 5:**
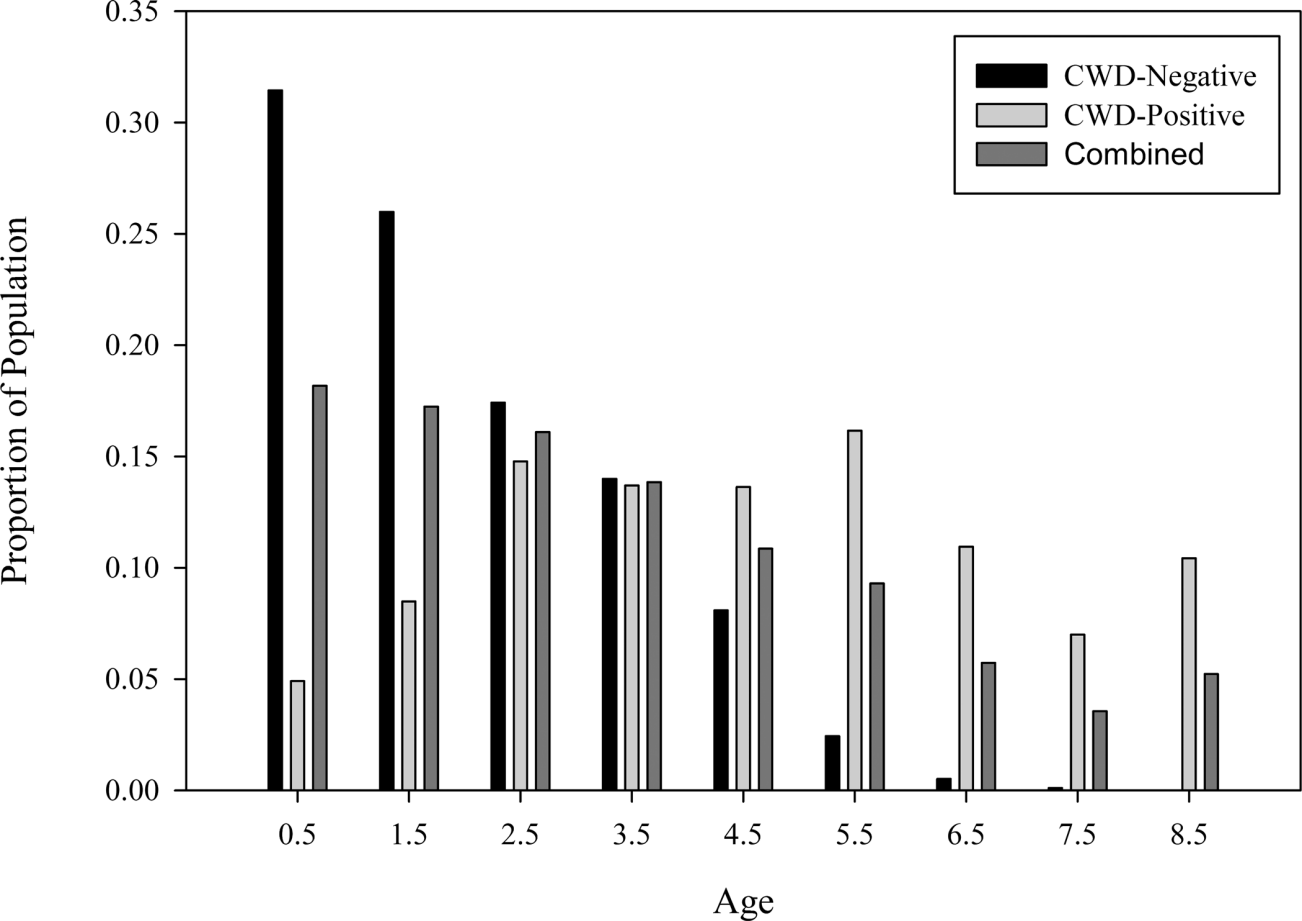
Population Age Structure. Proportional age structure of females separate by chronic wasting disease (CWD)-status and combined CWD-negative and CWD-positive deer of white-tailed deer captured, CWD-tested annually, marked with radio transmitter, and monitored by radio-telemetry SW of Glenrock, WY (2003–2010). Age structure was calculated from the dominant right eigenvector, w_1_, of the Leslie matrix population model of the transition matrix, **A**. The CWD-negative age structure was the result of assuming 0% CWD incidence modeled with a 9x9 **ANeg** transition matrix, the CWD-positive age structure was the result of assuming 100% CWD incidence with a 9x9 **APos** transition matrix, and the combined age structure was the result of modeling what is occurring in this population currently based on the 18x18 combined transition matrix, **A**; this result represents the actual population age structure.

The dominant right eigenvector, w_1_, determined the proportion of CWD-negative deer was highest in the fawn and yearling age classes and continued a constant, steep downward slope until the proportion was 0.005 in 6.5 years-old deer (Fig 5). Conversely, proportion of CWD-positive deer was lowest in fawns and yearlings, climbed to approximately 0.15 in 2.5 years-old deer and then plateaued (Fig 5). The age structure of all deer combined showed the majority of the population was found in the first three age classes followed by a rapid decline in older age classes (Fig 5), indicating the age structure was shifted to the left and dominated by young, immature, and sub-prime-aged deer.

## Discussion

The difference in survival by CWD-status and the high proportion of CWD-positive deer in this population help explain the declining population trend (*λ*_1_ = 0.896). The CWD-positive deer were 4.5 times more likely to die annually than CWD-negative deer. These results support concerns of wildlife managers, wildlife disease experts, and conservationists that this endemic (chronic) disease can negatively impact deer population sustainability at high disease prevalence. The sensitivity analysis and LTRE indicated survival of fawns, yearlings, and 2.5 year-old CWD-negative deer were primarily responsible for the reduction in *λ*_1_ caused by CWD. It is likely that CWD and hunter harvest, the main causes of mortality, have produced the young age structure observed in this population. At the current *λ*_1_, this population is not sustainable with possible extinction in 48 years at current levels of mortality and fecundity given the worst-case scenario of frequency dependent transmission [39] and no immigration or genetic selection for less susceptible genotypes for CWD [40].

Our estimate of *λ* is the lowest reported for a free-ranging cervid population with endemic CWD. Dulberger et al. [41] reported a *λ* of 0.97 (95% credible interval = 0.82–1.09) in a CWD-endemic mule deer population in Colorado, and *λ* = 1.0 has been reported for CWD-endemic elk populations in South Dakota and Colorado [42, 43]. These values were not particularly worrisome as *λ* either overlapped 1.0 given the credible interval or was equal to 1.0, indicating stable populations. It is particularly concerning how low our *λ*_1_ value was given that the study species was white-tailed deer, which have a higher lifetime reproductive potential than the other three CWD susceptible species.

Hunter harvest often is a major cause of mortality in white-tailed deer, which are the most common and wide-spread big game species in North America. We demonstrated that CWD-positive adults were over-represented in hunter harvest, and others [44] have suggested CWD-positive mule deer also are more vulnerable to hunter harvest. The behavioral shifts, including movement patterns, changes in breeding behavior during harvest, decreased reaction time to stimuli, and changes in habitat type used by CWD-positive mule deer may have caused biased harvest proportions. Conversely, Grear et al. [45] found no difference in harvest susceptibility between CWD-negative and CWD-positive white-tailed deer in Wisconsin, perhaps due to relatively low CWD prevalence (6.3% in adults). It is probable that the behavioral changes suggested by Conner et al. [44] affect CWD-positive deer susceptibility to harvest. Captive CWD-positive deer often show altered response to human activity [4], including an apparent lack of recognition of human presence. Activity analysis suggested CWD-positive bucks did not participate in the rut at the same level as CWD-negative bucks; the rut coincided with the hunting season [11]. Our data support the notion that CWD-positive bucks were less aware of the rut and the hunting season and were more susceptible to being shot by a hunter.

Over-representation of CWD-positive deer in the hunter harvest suggests behavior is altered by CWD prior to clinically recognizable CWD infection. Rather than thinking of CWD as a strictly pre-clinical disease followed by a short, obvious clinical stage of disease, we believe CWD infection should be envisioned as a slow, progressive decline in health and alteration of normal behavior, which ends with clinically recognizable disease. Given the relatively short clinical stage of CWD and the limited hunting season, it is hard to believe CWD-positive deer would be more susceptible to harvest if this slow alteration in health and behavior does not occur. Further, the majority of hunters do not intentionally harvest emaciated or sick animals.

There was a discrepancy in sex ratio of deer that died of clinical CWD (female: male = 12: 5). The high proportion of bucks in the harvest (76%) and over-representation of CWD-positive deer compared to CWD-negative deer may explain why females comprised 71% of clinical CWD cases. Data suggest CWD-positive bucks were harvested at a higher rate than expected and prior to reaching terminal stages of disease while the low harvest rate of does facilitated disease progression to clinical CWD. Females lived longer (137.2 weeks) after testing positive for CWD than bucks (107.4 weeks), which supports this argument. Also, the matriarchal social structure of females may explain why CWD incidence was higher in females and a more steady progression than males. Males were removed earlier in disease progression and had less time to spread disease directly to susceptible bucks in their bachelor herds throughout most of the year. Meanwhile, females progressed to clinical CWD, presumably shedding infectious prions into the environment and transmitting prions directly to susceptible females in their familial groups early in infection [46] and throughout most of the year. It is known that CWD prevalence is not spatially homogenous [47–50]. White-tailed deer are highly faithful to small home ranges in the Rocky Mountain West [11]. Prolonged prion shedding by CWD-positive does within their home range, including favored bedding locations, accompanied by communal grooming and shared home ranges with females provided opportunity for disease transmission through time.

Our study finding of higher incidence in does than bucks contradicts other reported studies that documented higher incidence in bucks than does (e.g., [45, 50, 51]). Presumably in hunted populations, bucks were the favored hunted sex as well. We believe that this discrepancy may be a function of the riparian habitat concentrating white-tailed deer and thus environmental contamination and allowing for the proposed role of does in the transmission of CWD in our study system. It is possible that in the future, when other habitats, such as winter lots in Wisconsin (where CWD has not been endemic for as long as Wyoming) have had similar time to become equivalently contaminated, does may become similarly important to transmission and incidence may increase in does in these population. In other words, perhaps our study population is an indicator of things to come, where initially bucks experience higher incidence until a threshold is met when does experience higher CWD incidence. This scenario assumes concentrated environmental contamination, however. For wide-ranging and dispersed populations, bucks may always experience higher incidence than females.

It is important to note that hunters may have had a bias in regards to harvesting collared deer. It is possible that hunters avoided shooting collared does in lieu of harvesting an uncollared doe to avoid altering the study results and to not have to deal with the hassle of returning a collar. Hunters targeting bucks may not have had such concerns if the antler size was large enough. If this was the case, then we may have over-emphasized the ratio of bucks to does in the harvest ratio. We believe this bias was relatively minor, at least within the main study site that encompassed the majority of the winter range, because hunters were forced to use one hunting outfitter on the VR Ranch and after conversations with this outfitter, they at least claimed to not be biased for or against harvesting collared animals.

Pregnancy and recruitment results indicate CWD does not compromise reproduction in female white-tailed deer. Blanchong et al. [52] also determined pre-clinical CWD did not negatively impact female reproduction in Wisconsin white-tailed deer. No difference in pregnancy indicates does participate in the rut regardless of CWD infection-status. It was not possible to determine if there was a difference in pregnancy and recruitment between pre-clinical and clinical CWD-positive does. However, it was common during the study to find one or two near-term fetuses in clinical-CWD female carcasses during the third trimester (Cornish and Edmunds, unpublished data). It is likely that fetuses exacerbate emaciation and hasten the death of does with terminal CWD. Our findings suggest does in pre-clinical disease give birth to fawns and are as successful at raising fawns to early September as CWD-negative does. Equal reproduction by CWD-positive does should dampen somewhat the negative effects of CWD on deer populations. Future research on neonate and young fawn survival is warranted, specifically to address the ability of CWD-positive white-tailed deer does to raise young to the age of population recruitment.

Pregnancy-specific protein B (PSPB) is not 100% accurate; Duquette et al. [53] documented 5 cases where white-tailed deer were found to be pregnant by trans-abdominal ultrasound but were deemed nonpregnant by PSPB. However, overall they found strong agreement between the two methods and recommended using either depending on the study objectives. We feel comfortable that the PSPB was an appropriate test, but it is possible that we underestimated pregnancy rates and therefore overestimated *λ*_1_, which already is extremely low for a white-tailed deer population. Considering the high pregnancy rates reported in this study, the impact on *λ*_1_ from inaccurate tests likely was minimal.

The modeling exercise that determined *λ*_1_ can be expected to be less than 1.0 (assuming other vital rates remain constant) at a prevalence of 27% suggests that as CWD in a population approaches these values, wildlife managers may choose to switch their objectives from lowering CWD prevalence by decreasing deer density to one of maintaining a sustainable population. The hunting-free Leslie matrix indicated removing additive hunting mortality in female deer resulted in a sustainable population. Therefore, it is recommended that at high CWD prevalence, hunting of does should be limited or ceased if the objective is to maintain population numbers. Currently this is a rare situation in most CWD endemic areas due to the relatively short period of time CWD has been present in most locations; this population should serve as an indication of what can happen at high prevalence when CWD has been endemic for an extended time period. Through time as prevalence rises in other endemic populations, more managers will be forced to make these choices if more effective management strategies or treatments are not developed. This recommendation is contingent on continued surveillance and monitoring of CWD in deer and elk populations in endemic areas as well as few or only minor public health concerns regarding CWD transmission to humans or livestock. Furthermore, if it becomes possible to accurately target and remove CWD-positive deer in a cost-effective manner, this management approach should be implemented in these populations where non-targeted culling is likely to be detrimental to population sustainability.

This population highlights the potential long-term negative outcome of endemic CWD to population sustainability and stresses the importance of preventing CWD from becoming endemic in a population, rather than attempting to manage it after the fact. Therefore, as previously suggested [43], the best management strategy remains minimizing movement of CWD to new areas.

## Supporting Information

S1 Fig Study Area Map. The study area, including Deer Creek drainage and VR Ranch southwest of Glenrock, Wyoming where deer were captured, tested for chronic wasting disease, marked with radio transmitters, and tracked by radio telemetry (2003–2010).
(TIF)

S1 Table Causes of Mortality. Cause of mortality by sex and chronic wasting disease (CWD)-status of white-tailed deer captured, CWD-tested annually, radio-collared, and monitored by radio-telemetry on Deer Creek Drainage, southwest of Glenrock, WY (2003–2010). The *χ*^2^ analysis determined if CWD-positive deer were over-represented in mortality data based on 23.8% average annual CWD-prevalence. Analysis was based on observed and expected total CWD-negative and CWD-positive deer. The CWD-unknown deer were excluded from analyses and sex was not considered.
(DOCX)

S2 Table Leslie Matrix Population Model Sensitivity and Elasticity. Sensitivity and elasticity analysis of the 18 x 18 transition matrix, **A**, for the Leslie matrix population model for a chronic wasting disease (CWD)-endemic white-tailed deer population captured, CWD-tested annually, radio-collared, and monitored by radio-telemetry SW of Glenrock, WY (2003–2010). Results presented by age class-specific survival (CWD-negative (-) and CWD-positive (+)), fecundity, and CWD incidence sensitivity and elasticity results. Age class-specific survival, fecundity, and CWD incidence were incorporated into transition matrix, **A.**
(DOCX)

S3 Table Pregnancy Data Used in Analyses. (XLSX)

S4 Table Recruitment Data Used in Analyses. (XLSX)

S5 Table Survival Data Used in Analyses. (XLSX)

S6 Table Incidence Data Used in Analyses. (XLSX)
