# Supplementary material for: Chronic Wasting Disease Drives Population Decline of White-Tailed Deer
Source: PLoS One. 2016 Aug 30;11(8):e0161127. doi: 10.1371/journal.pone.0161127 (PMC5004924; doi:10.1371/journal.pone.0161127)
Supplement: S1 Table — Cause of mortality by sex and chronic wasting disease (CWD)-status of white-tailed deer captured, CWD-tested annually, radio-collared, and monitored by radio-telemetry on Deer Creek Drainage, southwest of Glenrock, WY (2003–2010). The χ2 analysis determined if CWD-positive deer were over-represented in mortality data based on 23.8% average annual CWD-prevalence. Analysis was based on observed and expected total CWD-negative and CWD-positive deer. The CWD-unknown deer were excluded from analyses and sex was not considered. (DOCX) [file pone.0161127.s002.docx]

**S1 Table. Causes of Mortality.**

|  | CWD-Negative | | | CWD-Positive | | | CWD-Unknown | | |  |  |  |
| --- | --- | --- | --- | --- | --- | --- | --- | --- | --- | --- | --- | --- |
| Cause of Death | Female | Male | Sum | Female | Male | Sum | Female | Male | Sum | Total | $\chi_{1}^{2}$ | *P*-value |
| Hunter Harvest | 6 | 19 | 25 | 5 | 14 | 19 | 0 | 2 | 2 | 46 | 8.88 | 0.029 |
| Capture-related | 6 | 8 | 14 | 5 | 1 | 6 | 0 | 0 | 0 | 20 | 0.134 | 0.714 |
| Emaciation-CWD | --- | --- | 0 | 12 | 5 | 17 | 0 | 0 | 0 | 17 | --- | --- |
| Predation | 3 | 1 | 4 | 1 | 0 | 1 | 0 | 0 | 0 | 5 | 0.10 | 0.753 |
| Vehicle Collision | 2 | 1 | 3 | 2 | 1 | 3 | 0 | 0 | 0 | 6 | 1.03 | 0.311 |
| EHD/AHD | 3 | 2 | 5 | 0 | 0 | 0 | 1 | 0 | 1 | 6 | 0.537 | 0.464 |
| Caught in Fence | 0 | 2 | 2 | 0 | 0 | 0 | 0 | 0 | 0 | 2 | 0.001 | 0.974 |
| Drowning | 1 | 0 | 1 | 0 | 0 | 0 | 0 | 0 | 0 | 1 | 0.371 | 0.543 |
| Unknown Cause | 6 | 4 | 10 | 2 | 2 | 4 | 1 | 0 | 1 | 15 | 0.008 | 0.930 |
| Total |  |  | 64 |  |  | 50 |  |  | 4 | 118 | 24.65 | **<**0.001 |

EHD/AHD, epizootic hemorrhagic disease and adenovirus hemorrhagic disease

Cause of mortality by sex and chronic wasting disease (CWD)-status of white-tailed deer captured, CWD-tested annually, radio-collared, and monitored by radio-telemetry on Deer Creek Drainage, southwest of Glenrock, WY (2003-2010). The $\chi^{2}$ analysis determined if CWD-positive deer were over-represented in mortality data based on 23.8% average annual CWD-prevalence. Analysis was based on observed and expected total CWD-negative and CWD-positive deer. The CWD-unknown deer were excluded from analyses and sex was not considered.
