# Supplementary material for: Chronic Wasting Disease Drives Population Decline of White-Tailed Deer
Source: PLoS One. 2016 Aug 30;11(8):e0161127. doi: 10.1371/journal.pone.0161127 (PMC5004924; doi:10.1371/journal.pone.0161127)
Supplement: S2 Table — Sensitivity and elasticity analysis of the 18 x 18 transition matrix, A, for the Leslie matrix population model for a chronic wasting disease (CWD)-endemic white-tailed deer population captured, CWD-tested annually, radio-collared, and monitored by radio-telemetry SW of Glenrock, WY (2003–2010). Results presented by age class-specific survival (CWD-negative (-) and CWD-positive (+)), fecundity, and CWD incidence sensitivity and elasticity results. Age class-specific survival, fecundity, and CWD incidence were incorporated into transition matrix, A. (DOCX) [file pone.0161127.s003.docx]

**S2 Table. Leslie Matrix Population Model Sensitivity and Elasticity.**

|  | **Sensitivity** | | | | **Elasticity** | | | |
| --- | --- | --- | --- | --- | --- | --- | --- | --- |
|  | **Survival** | |  |  | **Survival** | |  |  |
| **Age** | **CWD (-)** | **CWD (+)** | **Fecundity** | **Incidence** | **CWD (-)** | **CWD (+)** | **Fecundity** | **Incidence** |
| Fawn | **0.280** | 0.007 | 0.000 | -0.346 | **0.240** | 0.006 | 0.000 | -0.032 |
| 1.5 | **0.269** | 0.027 | 0.156 | -0.144 | **0.234** | 0.012 | 0.062 | -0.042 |
| 2.5 | 0.154 | 0.072 | 0.127 | -0.015 | 0.161 | 0.024 | 0.050 | -0.005 |
| 3.5 | 0.086 | 0.041 | 0.132 | -0.027 | 0.089 | 0.045 | 0.053 | -0.014 |
| 4.5 | 0.043 | 0.080 | 0.086 | -0.010 | 0.037 | 0.044 | 0.034 | -0.007 |
| 5.5 | 0.012 | 0.067 | 0.051 | -0.003 | 0.010 | 0.037 | 0.020 | -0.002 |
| 6.5 | 0.003 | 0.044 | 0.029 | -0.001 | 0.002 | 0.025 | 0.012 | -0.001 |
| 7.5 | 0.001 | 0.026 | 0.017 | 0.000 | 0.000 | 0.015 | 0.007 | 0.000 |
| 8.5 | 0.000 | 0.034 | 0.021 | 0.000 | 0.000 | 0.019 | 0.008 | 0.000 |

Sensitivity and elasticity analysis of the 18 x 18 transition matrix, **A**, for the Leslie matrix population model for a chronic wasting disease (CWD)-endemic white-tailed deer population captured, CWD-tested annually, radio-collared, and monitored by radio-telemetry SW of Glenrock, WY (2003-2010). Results presented by age class-specific survival (CWD-negative (-) and CWD-positive (+)), fecundity, and CWD incidence sensitivity and elasticity results. Age class-specific survival, fecundity, and CWD incidence were incorporated into transition matrix, **A.**
